# Supplementary material for: αV Integrin Expression and Localization in Male Germ Cells
Source: Int J Mol Sci. 2021 Sep 2;22(17):9525. doi: 10.3390/ijms22179525 (PMC8431249; doi:10.3390/ijms22179525)
Supplement: Supplementary file 1 [file ijms-22-09525-s001.zip › ijms-1354875-SI.pdf]

αV integrin expression and localization in male germ cells

Veronika Palenikova[1,2,†] and Michaela Frolikova[1,†], Eliska Valaskova[1], Pavla Postlerova[1,3], Katerina Komrskova[1,4,*]

[1]Laboratory of Reproductive Biology, Institute of Biotechnology of the Czech Academy of Sciences, BIOCEV, Prumyslova 595, 252 50 Vestec, Czech Republic

[2]Department of Biochemistry, Faculty of Science, Charles University, Hlavova 8, 128 40 Prague 2, Czech Republic

[3]Department of Veterinary Sciences, Faculty of Agrobiology, Food and Natural Resources, University of Life Sciences Prague, Kamycka 129, 165 00 Prague 6, Czech Republic

[4]Department of Zoology, Faculty of Science, Charles University, BIOCEV, Vinicna 7, 128 44 Prague 2, Czech Republic

Supplementary table and figures

Supplementary Table S1. Characterization of the cell population of testicular elutriation fractions using specific gene markers for cell subtypes by qRT-PCR. Normalization was to Rps2 housekeeping gene. Data show relativity between cell populations and whole testes lysate; >1 is consider as strongly enriched fraction by the specified cell-type.

**Gene markers**	**Theoretical cell populations after elutriation**						**Primary source**
	Fraction 1	Fraction 2	Fraction 3	Fraction 4	Fraction 5	Testes	
C-kit	0.399	1.325	**3.807**	**4.306**	0.677	1	Spermatogonia
Sycp3	0.356	0.903	1.286	1.665	**1.029**	1	Primary spermatocytes
Acrv1	**1.520**	**2.244**	1.300	0.571	0.309	1	Round spermatids
Dbil5	0.943	0.975	0.447	0.239	0.202	1	Round/elongating spermatids
Cyp11a1	0.008	0.083	0.838	2.452	0.858	1	Leydig cells
Wt1	0.062	0.221	0.224	0.219	0.131	1	Sertoli cells

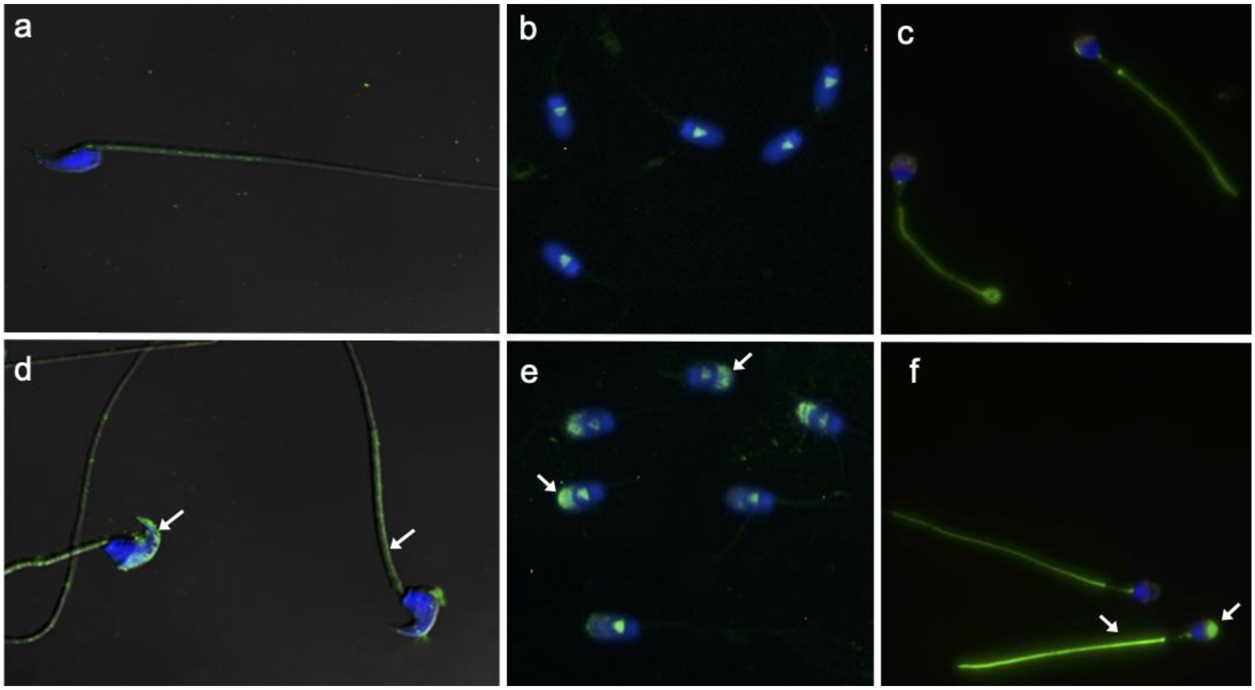

Supplementary Figure S1. Capacitated status of sperm evaluated by detection of protein tyrosine phosphorylation (pTyr). (a) mouse, (b) porcine and (c) human fresh, non-capacitated sperm display a low intensity pTyr signal (green). Contrary to in vitro capacitated (d) mouse, (e) porcine and (f) human sperm, which show visibly increased pTyr in the acrosome and/or tail (arrows). This pTyr increase indicates sperm reaching fully capacitated state. Nucleus is visualised by DAPI (blue).

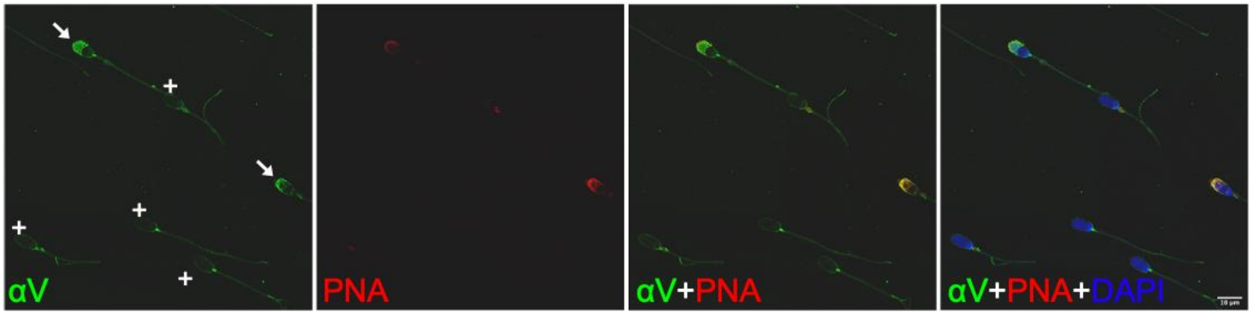

Supplementary Figure S2. Detection of αV integrin in acrosome-intact and acrosome-reacted porcine sperm. αV integrin (green) localization is present in the apical acrosome of sperm head in the acrosome-intact sperm (arrows). In contrary αV integrin is detected within the whole sperm head in the acrosome-reacted sperm (cross). Acrosome status is visualised by PNA lectin (red). Nucleus is stained by DAPI (blue). Scale bar represents 10 μm.

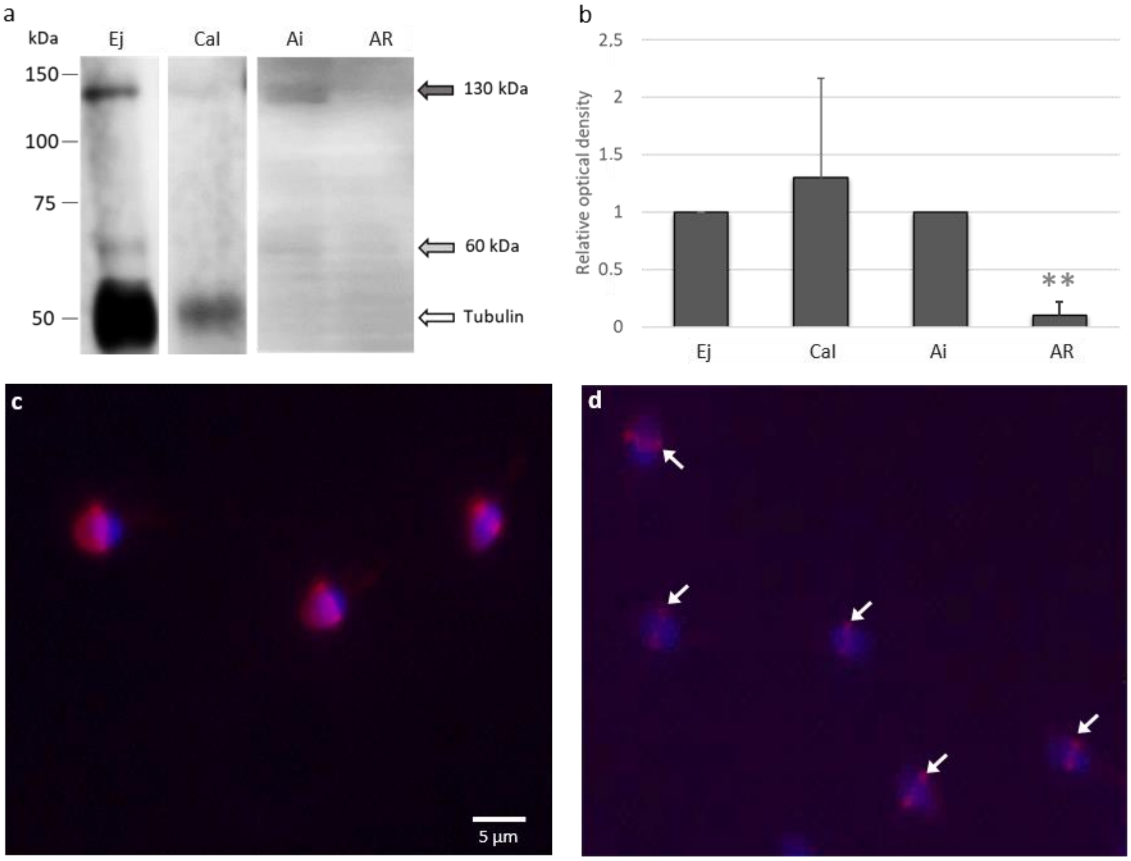

Supplementary Figure S3. Detection of αV integrin in human acrosome-intact and acrosome-reacted sperm. (a) Western blot immunodetection of αV integrin in protein extracts from following human sperm: ejaculated (Ej), after the acrosome reaction induced by calcium ionophore (CaI), acrosome-intact (Ai) and acrosome-reacted (AR) using sorting based on PNA labelling; (b) densitometry analysis of αV integrin in samples shows that CaI sperm contain both acrosome-intact and acrosome-reacted sperm; the acrosome-reacted sperm sample obtained after the sorting shows a decreased amount of αV integrin ($p < 0.02$; **). (c) PNA labelling (red) in acrosome-intact sperm, (d) PNA labelling in the equatorial segment (white arrows) of acrosome-reacted-sperm. Nucleus is visualised by DAPI (blue). Scale bar represents 5 µm.
